# TOILETING AND MOBILITY ASSISTANCE PREFERENCES OF PATIENTS ON AN ACUTE CARE FOR ELDERS (ACE) UNIT

**DOI:** 10.1093/geroni/igac059.2020

**Published:** 2022-12-20

**Authors:** Lucy Wilson, Emily Hollingsworth, Avantika Shah, Sandra Simmons

**Affiliations:** Vanderbilt University Medical Center, Nashville, Tennessee, United States; Vanderbilt University Medical Center, Nashville, Tennessee, United States; Johns Hopkins University, Baltimore, Maryland, United States; Vanderbilt University Medical Center, Nashville, Tennessee, United States

## Abstract

While the Agency for Healthcare Research and Quality (AHRQ) fall prevention guidelines include recommendations for providing consistent toileting and mobility assistance during hospitalization, little is known about hospitalized older adults’ preferences for receiving such assistance. The aim of this study was to identify older adults’ perceived need and preferences for toileting and mobility assistance during their hospital stay. We interviewed 150 patients aged 50 or older and asked about their perceived need for toileting and mobility assistance and how frequently hospital staff provided it. A total of 75 patients (50%) reported a need for toileting assistance; and, of those who reported a need for assistance, 95% reported that staff provided assistance at a frequency that met their needs. A total of 72 patients (48%) reported a need for mobility assistance; however, an additional 41 patients (27%) had not yet attempted to ambulate at the time of the interview. Across all patients, 100 (67%) reported either no attempt or having ambulated only once during their hospital stay. Most patients who required toileting or mobility assistance stated it was “very” or “extremely important” to receive assistance (82% and 76%, respectively). In summary, approximately one-half of older patients on an ACE unit require toileting and/or mobility assistance. Efforts to provide consistent toileting and mobility assistance during hospitalization could require significant staff time based on the number of patients who need it. Further research is needed to determine how to comprehensively implement AHRQ fall prevention guidelines in the hospital setting for older patients.

